# Development of an Experimental Model to Study the Relationship Between Day-to-Day Variability in Blood Pressure and Aortic Stiffness

**DOI:** 10.3389/fphys.2015.00368

**Published:** 2015-12-08

**Authors:** Camille Bouissou-Schurtz, Georges Lindesay, Véronique Regnault, Sophie Renet, Michel E. Safar, Vincent Molinie, Hubert Dabire, Yvonnick Bezie

**Affiliations:** ^1^Institut National de la Santé et de la Recherche Médicale, U955, Equipe 03Créteil, France; ^2^Cardiovascular Department, Institut de Recherches ServierSuresnes, France; ^3^Institut National de la Santé et de la Recherche Médicale, U1116Nancy, France; ^4^Groupe Hospitalier Paris Saint-Joseph, Department of PharmacyParis, France; ^5^Centre de Diagnostic et Université René Descartes, Hôtel-Dieu Hospital, UFR MédecineParis, France; ^6^Department of Pathology, Centre Hospitalier Universitaire La MeynardFort de France, France

**Keywords:** long-term blood pressure variability, diastolic blood pressure, aortic stiffness, pulse wave velocity, spontaneously hypertensive rats, telemetry, fibronectin

## Abstract

We aimed to develop an animal model of long-term blood pressure variability (BPV) and to investigate its consequences on aortic damage. We hypothesized that day-to-day BPV produced by discontinuous treatment of spontaneously hypertensive rats (SHR) by valsartan may increase arterial stiffness. For that purpose, rats were discontinuously treated, 2 days a week, or continuously treated by valsartan (30 mg/kg/d in chow) or placebo. Telemetered BP was recorded during 2 min every 15 min, 3 days a week during 8 weeks to cover the full BP variations in response to the treatment schedule. Pulse wave velocity (PWV) and aortic structure evaluated by immunohistochemistry were investigated in a second set of rats treated under the same conditions. Continuous treatment with valsartan reduced systolic BP (SBP) and reversed the aortic structural alterations observed in placebo treated SHR (decrease of medial cross-sectional area). Discontinuous treatment with valsartan decreased SBP to a similar extent but increased the day-to-day BPV, short term BPV, diastolic blood pressure (DBP), and PWV as compared with continuous treatment. Despite no modifications in the elastin/collagen ratio and aortic thickness, an increase in PWV was observed following discontinuous treatment and was associated with a specific accumulation of fibronectin and its α_v_-integrin receptor compared with both groups of rats. Taken together the present results indicate that a discontinuous treatment with valsartan is able to induce a significant increase in day-to-day BPV coupled to an aortic phenotype close to that observed in hypertension. This experimental model should pave the way for future experimental and clinical studies aimed at assessing how long-term BPV increases aortic stiffness.

## Introduction

Enhanced aortic stiffness is a significant and independent risk factor for all-cause of cardiovascular mortality, primarily coronary heart disease and stroke in man (Laurent et al., 2006). Despite the association between arterial stiffness and hypertension, it remains difficult to separate the causal effects of blood pressure (BP) elevation and changes in other associated haemodynamic parameters on arterial wall remodeling (Papadogiannis and Protogerou, 2011).

It is from this perspective that BP variability (BPV) has become a focus of attention. Several lines of evidence suggest that BPV is associated with increased cardiovascular risk independent of BP. Indeed, there is convincing evidence that BPV within 24 h is an independent predictor of the incidence of cardiovascular events in man (Mancia et al., 2007, 2012) and produces end-organ damage in a few experimental models (Van Vliet et al., 1999; Miao et al., 2003; Bouissou et al., 2014). Long-term visit-to-visit BPV increase, which potentially reflects a more diverse set of influences than short-term 24-h BPV, has also recently been described to independently predict clinical outcomes (Papadogiannis and Protogerou, 2011).

Results from clinical studies often conflictdue to the variety of ways to express BPV in man [the standard deviation of BP or its coefficient of variation and the average real variability (ARV) of BP], the period selected for the assessment of BPV (day, night, 24-h, or visit-to-visit periods) and the hemodynamic parameter evaluated (systolic or diastolic BP; Wei et al., 2014; Taylor et al., 2015).

Nevertheless, long-term intra-individual BPV has little correlation with concomitant short-term 24-h BPV and may result from different determinants. Seasonal changes due to ambient temperature and daylight hours, low patients' compliance and adherence to antihypertensive treatments or behavioral changes related to workdays and weekends are frequently cited as determinants of long term BPV (Mancia et al., 2012).

Important limitations of the current data from visit-to-visit BPV have been mentioned and related to the difficulty in quantifying precisely the magnitude of BP variations seen clinically and its repercussions on organ damage. Non-invasive ambulatory BP monitoring is the only approach suitable for large, long-term studies with BP readings spaced by less than an half hour to more precisely quantify the magnitude of the 24-h BP variations (Mancia, 2012). Since, these non-invasive automatic BP measurements require the patient to stop any activity at the time of measurement, this approach does not capture true BP variability in real-life conditions. Owing to the fact that people cannot practically wear an ambulatory BP cuff all the time, in real-life practice, it is also impossible to maintain the same frequency of BP measurements over long time periods.

Finally, information on the factors involved in long-term BP variations are scattered and incomplete. Little is known about the factors responsible for the BP differences that have been observed between visits spaced by months or years in observational studies and antihypertensive drug trials.

For all these reasons, the use of experimental models which allow BP monitoring and arterial stiffness measurement in conscious animals via telemetry and the full characterization of organ damage by *ex-vivo* methods seem to be an important step to identify the effects of day-to-day BPV enhancement on the cardiovascular system.

To our knowledge, no previous study has assessed vascular damage associated with isolated long-term BPV in experimental models. We addressed this issue in the development of an experimental model of day-to-day chronicle BPV increase based on administration of a discontinuous treatment of anti-hypertensive drug in spontaneously hypertensive rats (SHR). To achieve our goal, SHRs were treated with valsartan, an angiotensin II type 1 (AT1) receptor antagonist, at an antihypertensive dose, 2 days a week during 8 weeks (Lacolley et al., 2002). At the end of the treatment, SBP variations, long-term BPV, pulse wave velocity (PWV), and arterial structure and composition were investigated.

## Materials and methods

### Animals

Fifty-one male SHRs (Janvier, Le Genest St Isle, France) were received at 5 weeks of age and housed in a temperature and humidity controlled facility with a 12 h light/dark cycle. They were randomly assigned to a continuous treatment of placebo or valsartan (30 mg/kg/d; (Lacolley et al., 2002)), dosed pellets (Dietex France), or discontinuous treatment of valsartan (30 mg/kg/d, during 24 h, twice a week every Monday and Thursday). Treatment began at 8 weeks old and ended at 16 weeks old. All procedures were conducted in accordance with and approved by the Animal Ethics Committee of the “Ecole Nationale Vétérinaire d'Alfort” and conformed to the Guide for the Care and Use of Laboratory Animals, published by the National Institutes of Health.

Two distinct sets of rats were used for this study:

- Set 1: SHR implanted with a pressure transducer and used for characterization of BP and BPV (*n* = 5) for processing and analysis of telemetered hemodynamics
- Set 2: Rats devoted to PWV measurement and arterial structure analysis (*n* = 12).

### Blood pressure recording and arterial measurements

**Figure 1:**
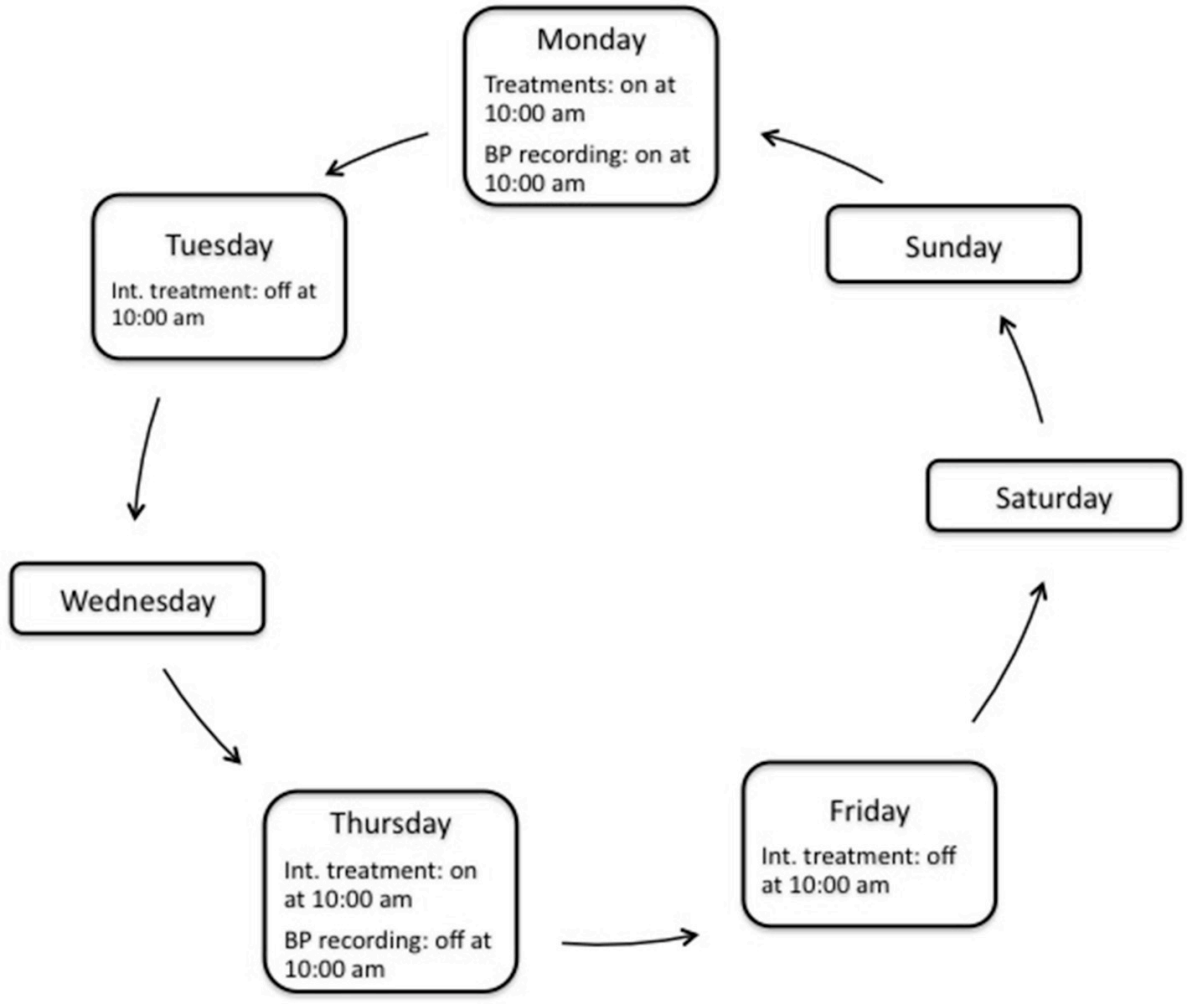
**Schematic representation of a week schedule of treatment and blood pressure (BP) recording**. The total duration of treatment was 8 weeks. Int. treatment, intermittent (i.e., discontinuous) treatment.

After 1 week of acclimation, 5 SHRs of each group (set 1) were equipped with a telemetric device as previously described (Basset et al., 2004). In brief, the rats were anesthetized with sodium pentobarbital (60 mg/kg, ip) and kept on a heating pad throughout the implantation of the BP telemeter (model TA11PA-C40; Data Sciences International, The Netherlands). The catheter of the BP telemeter was inserted into the abdominal aorta. The telemetric transmitter probe was positioned subcutaneously on the right flank. To reduce any infection and pain, the rats received amoxicillin (Clamoxyl; SmithKlineBeecham Laboratories, Nanterre, France; 20 mg/kg ip) and ketoprofen (Profenid; Aventis, Paris, France; 5 mg/kg ip). The rats were then housed in individual cages placed on the telemetric receivers, and allowed to recover over 2 weeks. At the end of the recovery period, treatments and BP recordings started according to the schedule displayed in Figure 1. Placebo treated (PT) and continuously treated (CT) groups received normal chows (0.25% NaCl, Dietex, Saint Gratien, France) and valsartan encapsulated chows, respectively, everyday throughout the treatment's duration. In discontinuously treated (DT) group, valsartan was given every Monday and Thursday at 10:00 am and replaced by normal chow every Tuesday and Friday at 10:00 a.m. The exposure to valsartan was thus 24 h twice a week every Monday and Thursday. The therapeutic withdrawal of 48 h was chosen to allow the complete elimination between the 2 days of administration (elimination half-time of Valsartan of 5–7 h for the rat). In the three groups of rats, BP was recorded every week from Monday 10:00 a.m. to Thursday 10:00 a.m., during 2 min every 15 min. The 2 min recordings were averaged to give a value of BP every 15 min yielding 4 BP values per hour and 288 BP values for the 3 days recording. For each animal, a mean value of systolic and diastolic BP was calculated each week as the mean of the 288 SBP values determined over the 3 days recording.

In a second set of experiments, 12 unequipped SHRs from each group were used for the measurement of PWV as previously described (Cosson et al., 2007). PWV was performed at the end of the experiments, the third day of the eighth week. At the end of the treatment, under pentobarbital anesthesia (60 mg/kg, ip), one catheter was inserted into the lower abdominal aorta via the left femoral artery; another catheter was introduced, through the left common carotid artery, into the descending aorta. Proximal and distal aortic BP signals were simultaneously recorded on line (Chart version 5.2; AD Instruments) and stored on a microcomputer (PowerMac 4400/200; Apple) for further analysis. At the end of the recording, the rats were euthanized by an overdose of sodium pentobarbital. After dissection, the tips of the two catheters were visually marked, and the distance between them was carefully measured three times; the mean of which was used. For PWV measurement, the foot-to-foot method was used to determine the time delay between the proximal and the distal aorta (Cosson et al., 2007). The aorta and the heart were then removed for evaluation of end organ damage.

### Investigation of blood pressure variability

The spectral analysis of short-term BPV and inter-beat interval (IBI) was computed with a fast Fourier transform and quantified in terms of low frequency (LF) and high frequency (HF) spectral power of SBP and IBI, calculated between 0.2–0.6 and 1.0–2.5 Hz, respectively (Cerutti et al., 1991).

Short term BPV was also characterized by the mean ARV, a clinical index calculated by the following formula which evaluates the variability between consecutive and validated readings of BP (Mena et al., 2005; Hansen et al., 2010):

$$ARV=\frac{1}{\sum w}\sum_{k\;=\;1}^{n}w\times \left|BP_{k}-BP_{k-1}\right|$$

where, *k* ranges from 1 to *n, w* is the time interval between *BP*_*k*−1_ and *BP*_*k*_, and n is the number of BP readings in 24 h.

As ARV was never used, to our knowledge, in experimental models, we first evaluated this index in sino-aortic denervated (SAD) rats, a relevant model of increased short term BPV. For that purpose, we used telemetric BP data already published (Van Vliet et al., 1999). BP data were averaged by sequences of 2 min every 15 min to verify that ARV of SAD rats is increased in conditions of measurements close to those made within our experiments.

Daily variability was evaluated through the standard deviation (SD) of the 96 individual values of BP average of 15 min periods over the course of the 24 h recording. Calculation was performed on the third day of telemetric recording during the eighth week. As animals were subjected to a controlled 12 h light/dark cycle, calculation of the weighted 24 h SD, which is the mean of daytime and nighttime values weighted by the duration of each time period, was useless in the present study (Bilo et al., 2007).

To evaluate day-to-day variability i.e., long-term variability, the 3 days recordings of each week were analyzed separately using Chronos-Fit® software (Zuther et al., 2009). For each day, we measured the mean of the SBP and DBP. The long-term SBP variability was evaluated weekly as the SD of the three daily means. This method may be compared to the visit-to-visit variability used to evaluate long-term variability in clinical studies (Rothwell et al., 2010; Muntner et al., 2011).

### Biological characterization of the vascular endothelium

Plasma was collected before the rats were euthanized. Vascular endothelium was evaluated through quantification of von Willebrand factor (vWF) antigen and sCD146, a marker of the endothelial junctions (Lagrange et al., 2014). vWF was measured by ELISA (Asserachrom; Diagnostica Stago, Asnière-sur-Seine, France). Soluble CD146 was measured in plasma using a homemade ELISA as already reported (Lagrange et al., 2014). Briefly, costar high-binding 96-well plates (Corning, Corning, NY, USA) were coated overnight at 4°C with 100 μl of the anti-CD146 P1H12 clone at 1 μg/ml in carbonate-bicarbonate buffer (0.05 M, pH 9.6). Plates were blocked overnight with 125 μl of PBS containing 0.5% gelatin. Samples (100 μl) were assayed at 2 dilutions (1:5 and 1:10) in PBS containing 0.05% Tween and incubated for 2 h at room temperature. After subsequent revelation with the anti-CD146 2Q401 monoclonal antibody at 1 μg/ml for 1 h and horseradish-conjugated streptavidin at 1:1000 for 15 min at room temperature, color was developed with tetramethylbenzidine/H_2_O_2_ substrate. Absorbance was measured in a microplate reader at 450 nm.

### Determination of arterial structure and composition

Arterial structure was determined and quantified in 4% formaldehyde-fixed thoracic aorta extracted from the rats. The tissue was extracted from individual paraffin blocks and then inserted into a preformed paraffin recipient block (Tissue-Tek Quick-Ray System, Sakura Finetek France). The finished block was then cut into thick sections and mounted on Superfrost plus slides and subjected to independent tests.

Collagen, elastin, and media cross-sectional areas (MCSA) were quantified by morphological analysis after a Sirius red, orcein and HES (Hematoxillin, Eosin, Safran) staining, respectively, as previously described (Isabelle et al., 2015).

For immunohistochemical analyses, the following antibodies were used: a rabbit anti-integrin alpha5 and a rabbit anti-integrin alphaV (Chemicon International) and a mouse monoclonal anti-fibronectin (ab2040, Millipore). Immunohistochemistry was performed on 4 μm sections. Heat-mediated antigen retrieval was performed in EDTA buffer pH 9 in water bath for 30 min. Immunostaining was performed on a Dako autostainer using a peroxidase-labeled polymer-based detection system (Envision plus, Dako) and diaminobenzidine as a chromogen. No specific staining was observed when primary antibody was omitted from the protocol (negative control). The distribution and quantification of staining were determined by computer-directed color analysis performed with the Quant'Image software (Quancoul, Talence, France; Bézie et al., 1998; Bouissou et al., 2014).

### Cardiovascular damage

Cardiac alteration was evaluated by the heart weight and by the measure of the Brain Natriuretic Peptide (BNP), a marker of heart failure. Plasma was collected before the rats were euthanized to measure the BNP with an Elisa method (AssayMax Rat BNP-32 ELISA Kit, Assaypro, St Charles, USA).

### Statistical analysis

All data are expressed as means ± SEM. One-way ANOVA followed by a Fisher PLSD was used to assess the significance of the results. Differences were considered significant at values of *P* < 0.05.

## Results

### Changes in blood pressure in conscious rats

The daily changes in BP in each group of treatment regimen was analyzed by comparing the daily mean SBP and DBP at the 8th week treatment (Table 1). Compared to PTgroup, both treatments with valsartan significantly reduced SBP during the 3 days recording. Whilst the effect on SBP appears reduced in DT compared to CT rats, no significant difference was observed between the two groups.

**Table 1:** **Telemetered hemodynamic changes in SHR after 8 weeks of treatment by valsartan**.

**72 h Means**	**Placebo**	**Continuous treatment**	**Discontinuous treatment**
Heart rate, beats min^−1^	319±4	316±3	327±4
Systolic blood pressure, mmHg	160.3±5.6	132.8±7.0^*^	141.3±5.5^*^
Diastolic blood pressure, mmHg	114.3±3.3	93.3±2.0^*^	105.6±2.5^*^^†^
Mean blood pressure, mmHg	129.6±2.5	106.4±3.3^*^	117.5±2.1^*^^†^
Pulse pressure, mmHg	46.0±3.4	39.6±4.4^*^	35.7±4.7^*^

* *p < 0.05 vs. placebo*;

† *p < 0.05 vs. continuous treatment. The values represent the average of the data acquired during 3 days of recording. Continuous treatment: administration of valsartan (30 mg/kg/d) during the 8 weeks of the experiments. Discontinuous treatment: administration of valsartan (30 mg/kg/d) twice a week (every Monday and Thursday) during the 8 weeks of the experiments*.

In contrast, the DBP decrease observed after continuous administration of valsartan was less effective in the DT group, so that DBP was slightly increased in DT vs. CT treatment by valsartan rats (Table 1).

**Figure 2:**
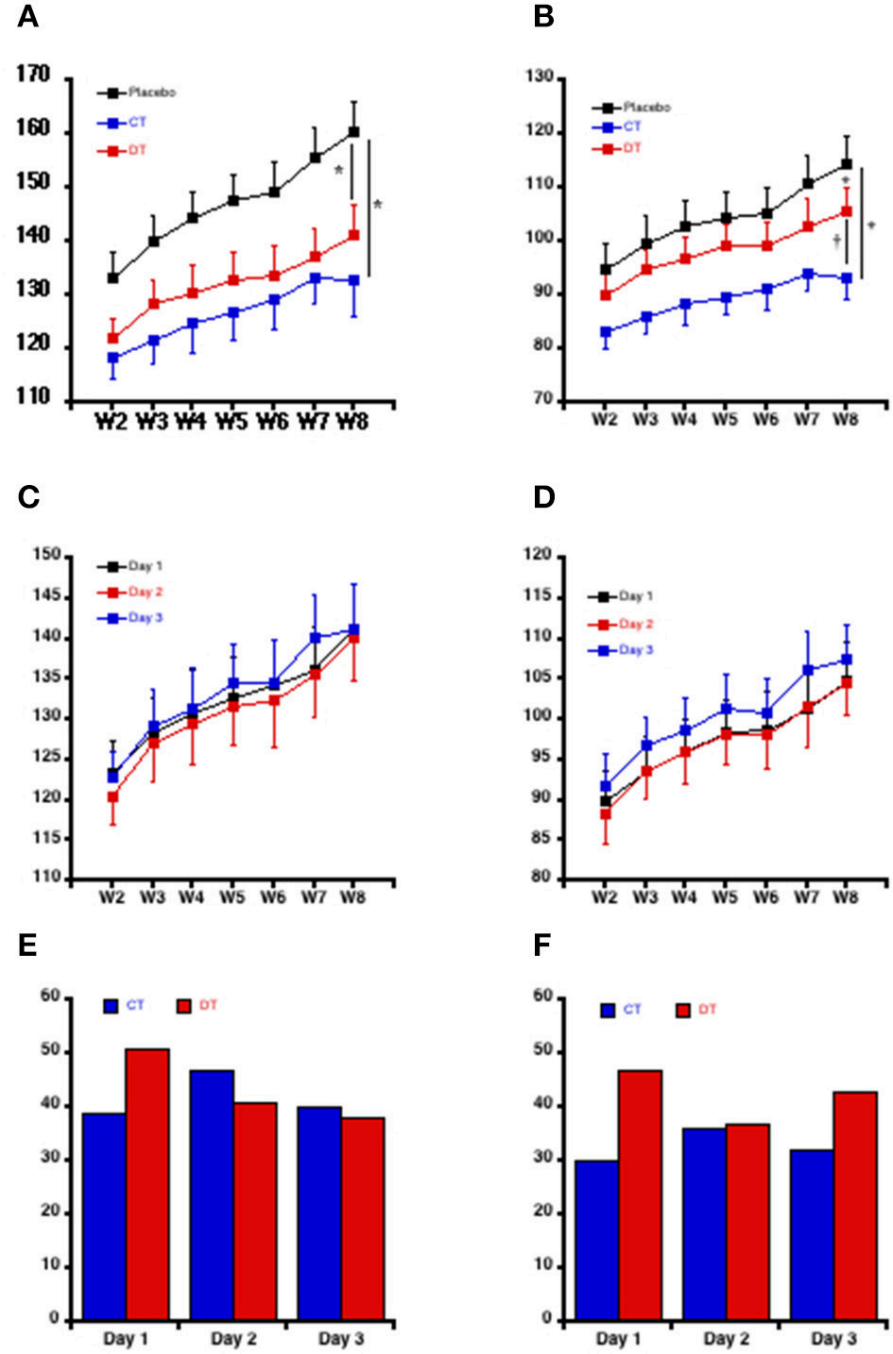
**Time-course evolution of the blood pressure in conscious rat**. Systolic **(A)** and diastolic **(B)** blood pressure during the 8 weeks study. Each point is the mean ± SEM of five rats. For each week and for each rat, the mean of 288 BP values was calculated (four values of BP per hour during 3 days recording). ^*^P < 0.05 vs. placebo; ^†^*P* < 0.05 vs. continuous treatment. The inter-day evolution of blood pressure during the 8 weeks study in response to the discontinuous administration of Valsartan is showing for the systolic **(C)** and diastolic **(D)** mean blood pressure. Each point is the mean ± SEM of the five rats of the discontinuous group. Day 1: Day of administration of valsartan. Day 2 and Day 3: days of Valsartan's withdrawal. The within-day variation of systolic **(E)** and diastolic **(F)** blood pressure is expressed through the difference between the first and the fourth quartile of blood pressure for the continuously (CT) and the discontinuously (DT) treated rats with valsartan.

The time-course changes in BP in the three groups of conscious rats are displayed in Figure 2. Compared to placebo-treated rats, both continuous and discontinuous treatments by valsartan significantly reduced SBP to a similar extent. The reduction of the SBP was significant from the beginning of the data acquisition, the second week of the experiments (Figure 2A). In contrast, DBP was significantly less reduced in DT than in CT SHRs (Figure 2B). As a function of the rats getting older, HR decreased with time and similarly in the three groups (results not shown).

In the DT group, the increase of DBP was obvious each week on the third day (day3) of BP telemetered follow-up, i.e., after 2 days of withdrawal of the valsartan (Figure 2D). In contrast, SBP was only slightly increased on the third day for the same rats (Figure 2C). These results were confirmed after analysis of the within-days differences between the first and the fourth quartiles of BP. Whilst the difference between the first and the fourth quartiles of SBP was increased in DT vs. CT rats on the first day of follow-up (i.e., when valsartan was being given for the discontinuous group after 2 days of withdrawing), SBP fell and reached the same values the second and the third days of follow-up after rechallenge of valsartan, indicating a persistent lowering of the SBP (Figure 2E). In contrast, even if DBP seems controlled by valsartan on the second day, its values were strongly increased in DT vs. CT rats during the third day (Figure 2F). Together, these data confirm that SBP and DBP patterns were different according to the treatment schedule.

### Blood pressure variability in conscious rats

**Figure 3:**
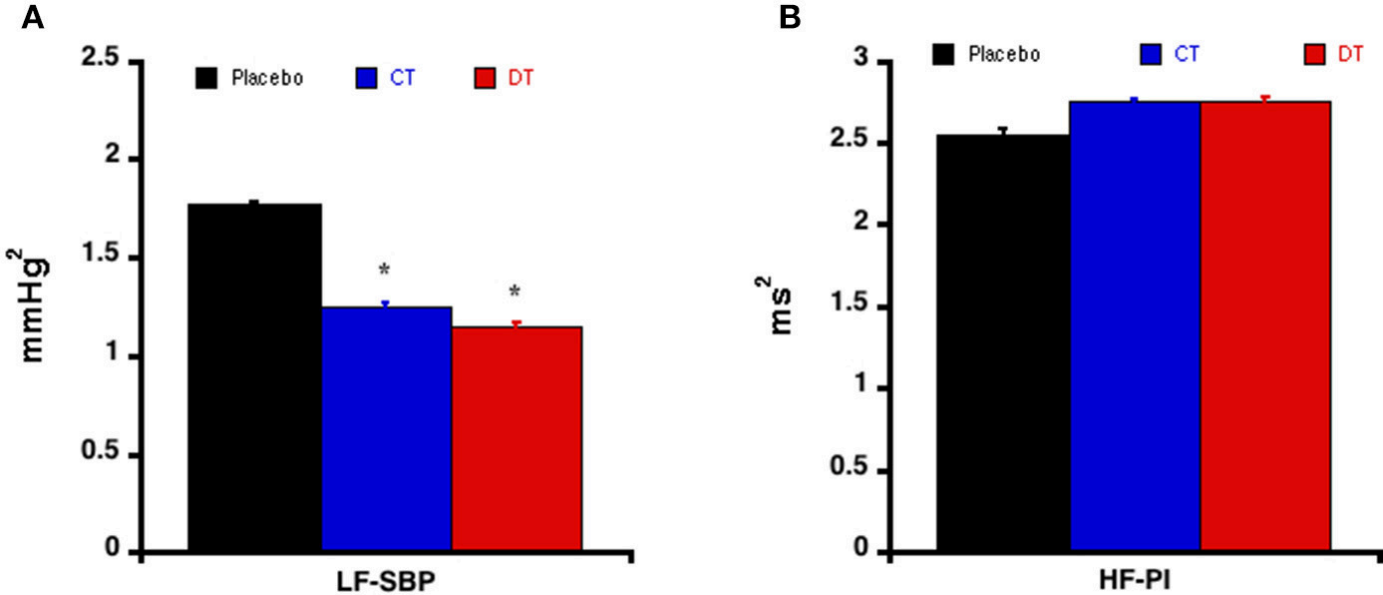
**Short term variability evaluated by spectral analysis**. Modifications of the low frequencies of systolic blood pressure **(A)** and high frequencies of interbeat interval **(B)** in response to the different treatments schedules. Each bar is the mean ± SEM of five rats. ^*^P < 0.05 vs. placebo.

The short-term variability of SBP expressed by the low frequencies of the SBP power spectrum was significantly decreased by both continuous and discontinuous treatments in accordance with the reduction in SBP (Figure 3), suggesting that valsartan reduced the activity of the sympathetic nervous system.

Table 2 indicates that short term BPV, expressed by ARV is increased, as expected, in SAD rats. Short-term systolic and diastolic BPV, expressed by ARV and the SD, were reduced by valsartan given continuously (Table 3). Nevertheless, discontinuous treatment by valsartan was unable to reduce short-term BPV in the same extent. Indeed both systolic and diastolic ARV and diastolic SD were increased compared to continuous treatment, and comparable to the placebo level 2 days following the withdrawal of valsartan at the end of the experiment (Table 3).

**Table 2:** **Telemetered hemodynamic changes and their variability in sinoaortic denervated rats**.

	**SAD (*n* = 6)**	**Sham (*n* = 6)**	***P*-value**
**24 H MEANS**			
Heart rate, beats min^−1^	400.0±7.8	398.2±8.0	NS
Systolic blood pressure, mmHg	133.9±2.9	129.3±1.7	NS
Diastolic blood pressure, mmHg	96.4±2.6	91.1±1.0	NS
Pulse pressure, mmHg	33.3±4.7	34.6±2.8	NS
**24 H SYSTOLIC BLOOD PRESSURE VARIABILITY**			
10-min SD, mmHg	10.0±0.8	8.2±0.5	NS
ARV, beats min^−1^	7.2±0.6	4.7±0.4	< 0.005
**24 H DIASTOLIC BLOOD PRESSURE VARIABILITY**			
10-min SD, mmHg	9.3±1.0	7.9±0.6	NS
ARV, mmHg	6.7±0.8	4.2±0.4	< 0.050
**24 H PULSE PRESSURE VARIABILITY**			
10-min SD, mmHg	1.6±0.2	1.6±0.2	NS
ARV, mmHg	1.2±0.2	1.0±0.1	NS

*SAD, Sinoaortic denervated rats; Sham, Sham-operated rats; Total SD, Mean standard deviation of values within 144 individual 10 min periods over the course of the 24 h recording; ARV, Average real variability of 24-h*.

**Table 3:** **Blood Pressure Variability in SHR in response to the different treatments schedules**.

	**Placebo**	**Continuous treatment**	**Discontinuous treatment**
**DIASTOLIC BLOOD PRESSURE**			
Mean DBP	113.7±3.5	93.1±2.2^*^	107.4±2.3^*^^†^
**SHORT TERM DBPV**			
Total SD 24 h	10.8±0.8	8.8±0.1^*^	11.3±1.1^†^
ARV	8.6±0.8	7.2±0.3^*^	8.6±0.4^†^
**DAY-TO-DAY DBPV**			
Total SD	0.55±0.27	0.84±0.17	2.29±0.42^*^^†^
CV	0.47±0.22	0.91±0.20	2.14±0.40^*^^†^
**SYSTOLIC BLOOD PRESSURE**			
Mean SBP	160.3±1.0	132.8±1.1^*^	141.3±2.6^*^
**SHORT TERM SBPV**			
Total SD 24 h	12.2±0.6	9.3±0.2^*^	9.7±0.5^*^
ARV	10.1±0.9	7.5±0.3^*^	9.1±0.4^†^
**DAY-TO-DAY SBPV**			
Total SD	1.03±0.36	1.10±0.25	2.66±0.40^*^^†^
CV	0.64±0.20	0.85±0.18	1.87±0.25^*^^†^

*Values are mean ± SEM*.

* p < 0.05 vs. placebo and

† *p < 0.05 vs. Continuous treatment. Continuous treatment: administration of valsartan (30 mg/kg/d) during the 8 weeks of the experiments. Discontinuous treatment: administration of valsartan (30 mg/kg/d) twice a week (every Monday and Thursday) during the 8 weeks of the experiments. DBPV, Diastolic blood pressure variability; SBPV, Systolic Blood Pressure Variability*.

**Figure 4:**
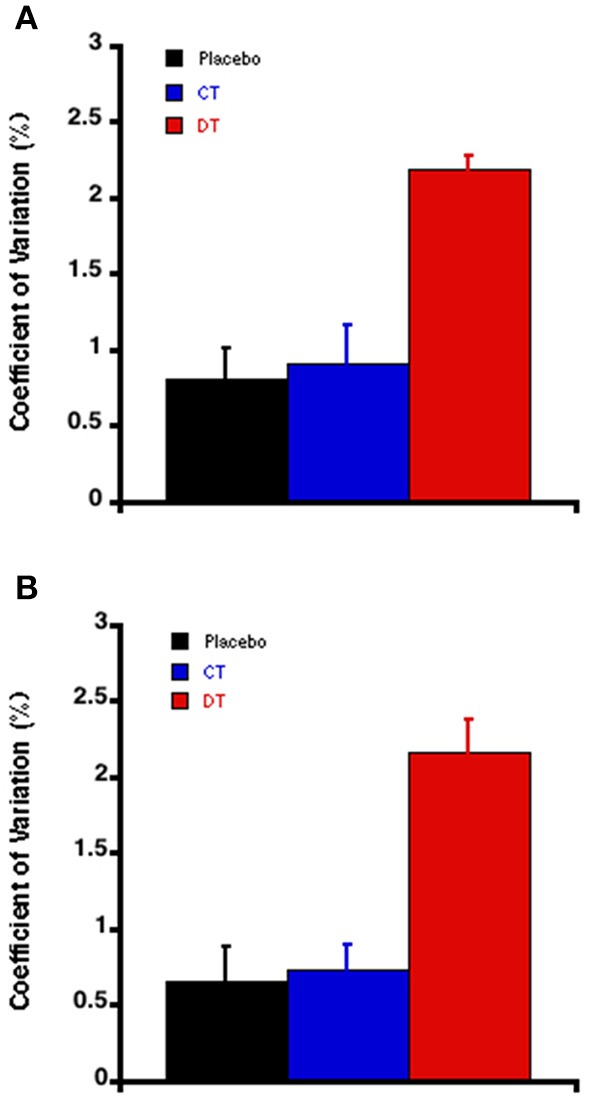
**The Day-to-day variability of diastolic blood pressure (A) and systolic blood pressure (B) estimated via their coefficient of variation at the end of the experiments (the 8 week)**. Each bar is the mean ± SEM of five rats. ^*^P < 0.05 vs. placebo; ^†^*P* < 0.05 vs. continuous treatment.

In contrast to short-term and daily BPV, day-to-day BP instability, expressed as the SD over 3 days recording, was significantly increased by discontinuous treatment with valsartan, compared to PT and CT groups (Table 3). This result wasn't related to the BP level as the average day-to-day SBP and DBP coefficient of variation were not different between the PT and the CT rats, these indexes were markedly higher after the discontinuous treatment by valsartan (Table 3; Figure 4).

### Aortic stiffness in anaesthetized rats

As in conscious rats, BP was significantly reduced to a similar extent in both CT and DT groups after the anesthesia (Table 4). Compared to PT rats, PWV was significantly reduced by a continuous treatment by valsartan, but remained unchanged in DT group compared with PT rats (Table 4). Similar results were obtained when PWV was normalized by DBP (beta index).

**Table 4:** **Pulse wave velocity in response to 8 weeks of treatment by valsartan in Spontaneously Hypertensive rats**.

	**Placebo (*n* = 7)**	**Continuous treatment (*n* = 7)**	**Discontinuous treatment (*n* = 5)**
Systolic blood pressure, mmHg	208±4	183±5^*^	190±10^*^
Diastolic blood pressure, mmHg	167±3	148±3^*^	150±6^*^
Mean blood pressure, mmHg	187±3	166±3^*^	169±7^*^
Pulse pressure, mmHg	41±2	35±2	40±4
Heart rate, bpm	336±11	344±11	347±5
Pulse wave velocity, cm/s	826±46	691±18^*^	751±22^†^
Beta Index (10^3^)	8.8±0.9	6.9±0.4^*^	8.0±0.4^†^

* P < 0.05 vs. placebo and

† *P < 0.05 vs. continuous treatment*.

### Cardiovascular damages

A continuous treatment by valsartan significantly reduced heart weight, while a discontinuous treatment does not (Table 5). However, BNP plasmatic levels remained unchanged in the three groups of rats (0.21 ± 0.02 mg/L for Placebo vs. 0.18 ± 0.01 and 0.19 ± 0.03 for CT and DT groups, respectively).

**Table 5:** **Effects of valsartan treatments on the weights of various organs**.

	**Placebo *n* = 8**	**Continuous treatment *n* = 8**	**Discontinuous treatment *n* = 8**
BW, g	370±9	374±6	385±7
Heart, g	1.52±0.03	1.37±0.03^*^	1.49±0.02^†^
Heart:BW, mg/g	0.42±0.01	0.37±0.01^*^	0.39±0.01
Left ventricular, g	1.10±0.03	1.02±0.02^*^	1.07±0.01
Kidney, g	2.66±0.04	2.64±0.04	2.71±0.03
Kidney:BW, mg/g	7.3±0.2	7.1±0.1	7.1±0.02

Body Weight, BW; The organ: BW ratios (mg/g) were based on the BW measured before organ extraction. Values are mean ± SEM;

* p < 0.05 vs. Control;

† *p < 0.05 vs. continuous treatment*.

Plasma von Willebrant factor (vWF) was increased in PT compared with both treated groups (Table 6), indicating that valsartan preserved endothelium integrity. In contrast, no difference was found for sCD146 antigen, indicating that endothelial junctions were not influenced by the treatments.

**Table 6:** **Aortic structure and composition**.

	**Placebo**	**Continuous treatment**	**Discontinuous treatment**
MCSA, 10^3^ μm^2^	163.9±0.9	152.6±1.9^*^	161.7±1.1^†^
Elastin, %	31±1.2	30±0.9	33±0.8
Collagen, %	14±0.9	12±2.4	15±1.0
Fibronectin, %	14.3±3.1	9.3±3.3	27.1±5.5^†^
Integrin α5, %	4.6±2.3	2.8±0.7	4.4±1.2
Integrin α v, %	2.5±0.8	2.2±0.8	6.5±2.4^*^^†^
VW factor, ng/mL	329.3±14.6	287.3±8.3^*^	268.8±13.0^*^
sCD146, OD_450nm_	0.49±0.09	0.43±0.05	0.34±0.02

Mean ± SEM of 12 rats. MCSA, media cross-sectional area;

* P < 0.05 vs. placebo;

† *P < 0.05 vs. continuous treatment*.

**Figure 5:**
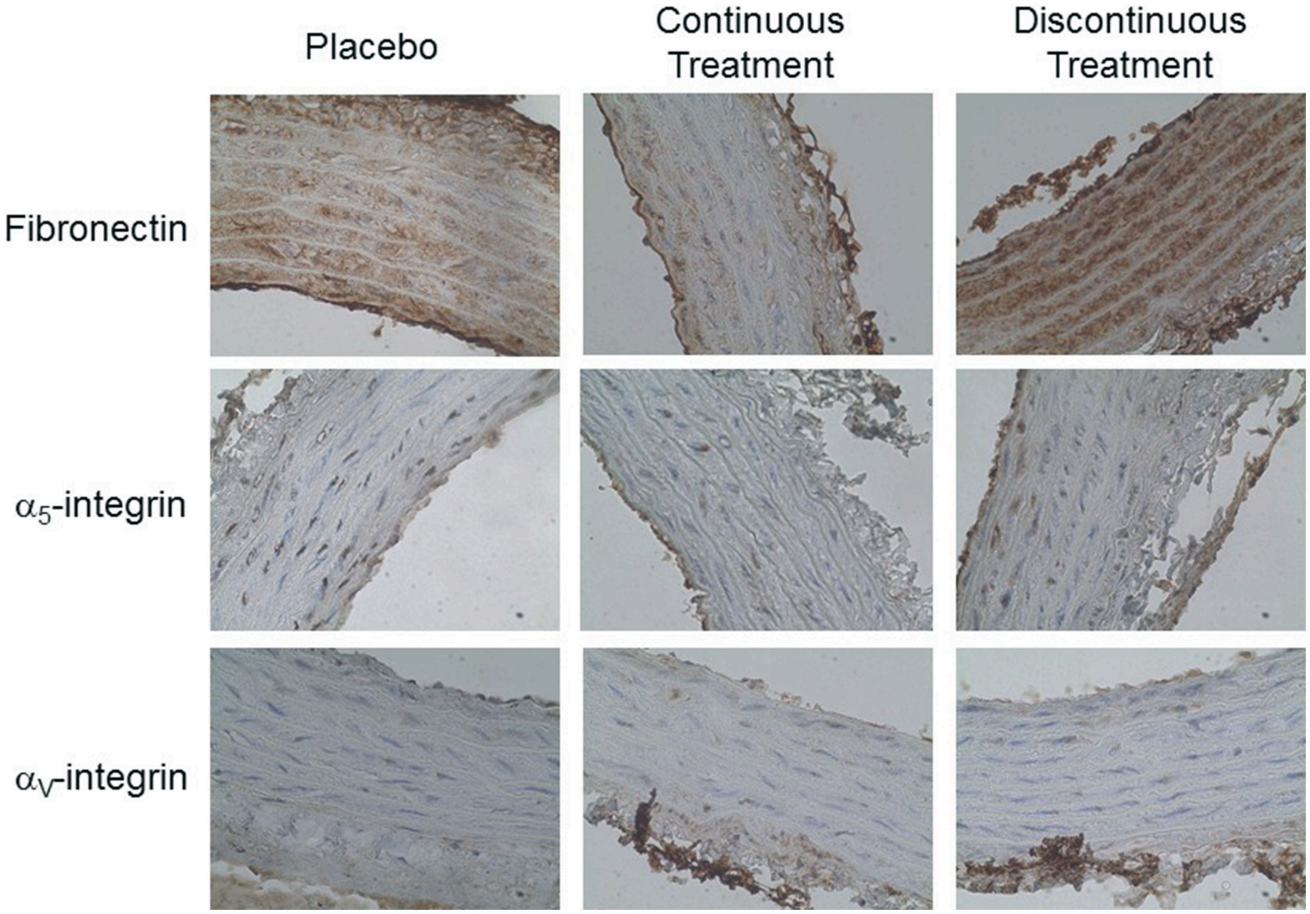
**Effect of discontinuous treatment by valsartan on abdominal aorta morphological changes examined by adhesion proteins staining (x40)**.

Immunohistochemical and biochemical characteristics of aortic structure appear in Table 6. Only the continuous treatment by valsartan significantly reduced medial wall cross-sectional area. Vascular collagen and elastin contents and density (Table 6; Figure 5) as well as size and number of nuclei of smooth muscle cells (data not shown) remained unchanged in the three groups. We observed that fibronectin content was significantly reduced by continuous treatment as compared to the PT group. In contrast, fibronectin accumulation was increased in DT group compared with the two other groups (PT or CT rats; Table 6). This accumulation of fibronectin was coupled to a specific increase of its α_v_-integrin.

## Discussion

Using a pharmacological approach to develop an animal model of long-term BPV, we have shown that discontinuous treatment with valsartan, twice a week, is associated with vascular damage in SHR. This induced change in day-to-day BPV was also associated with an increase in DBP and PWV, vascular hypertrophy, and fibronectin accumulation compared to continuous treatment with valsartan at the same dosage. These changes occurred despite similar SBP levels between the two groups.

Cross-sectional studies have shown a strong association of aortic stiffness with age and with cardiovascular risk factors such as hypertension, obesity, impaired glucose tolerance, and dyslipidemia (Mitchell et al., 2007). Other hemodynamic parameters like BPV are also able to reduce arterial elasticity (Parati et al., 1987; Laurent et al., 2006). The concept that the degree of variability of BP during a 24 h period bears a relation to organ damage that is independent of the BP level comes from animal research (Van Vliet et al., 1999; Su, 2006; Bouissou et al., 2014). Even if preclinical studies remain an essential way to explore and understand the relationship between arterial stiffness and BPV, the inherent cardiovascular risk of long term BPV has, to our knowledge, never been evaluated through pharmacological models. To meet this aim, we used an AT_1_ blocker (valsartan) with short half-time (6–9 h) twice a week, every Monday and Thursday, hypothesizing that arterial pressure would not be controlled on the days for which no treatment was given, thus inducing day-to-day BPV. Lowering of BP following drug withdrawal after inhibition of the renin-angiotensin system in SHR has been performed previously (Paull and Widdop, 2001; Woolard et al., 2003). These studies reported a persistant reduction in BP following drug withdrawal coupled with persistant cardiac hypertrophy a few weeks later. BPV wasn't evaluated, but the absence of hypertensive rebound following drug withdrawal in these previous studies suggests that day-to-day variability was marginal. More recently, Susic et al. reported that in another hypertensive rat model of partial adherence to antihypertensive therapy, arterial pressure decreased and reached control values (Susic et al., 2008), but once again, day-to-day variability wasn't explored. Thus, to our knowledge, our present experimental work is the first to report a strong day-to-day variability in hypertensive rats following discontinuous AT_1_ blocker treatments.

Telemetric BP recordings were obtained on three continuous days each week to cover the full BP fluctuations in response to the peak and trough concentrations of valsartan according to the treatment schedule. Interestingly, we found a clear dissociation between diastolic and systolic BP levels in response to discontinuous drug administration compared with continuous treatment. The time course evolution of systolic and diastolic BP levels showed that isolated DBP increased within 2 weeks following the commencement of the discontinuous drug regimen. In man, epidemiological observations have long reported that DBP is the strongest predictor of cardiovascular outcomes below 50 years of age, and with advancing years, a gradual shift from DBP to SBP and then to pulse pressure (PP) are the better predictors of coronary heart disease (Franklin et al., 2001; Li et al., 2014). DBP reflects the resistance the heart must overcome to eject blood. Increases in DBP therefore, reflects an increase in peripheral arterial resistance, which in turn might move reflection sites of the forward BP wave to more proximal locations, such that the reflected waves return earlier, thereby augmenting central SBP (Liu et al., 2013). In our present work, the cardiac mass (body-weight ratio) and plasmatic BNP were not different between both treated groups of rats indicating that the increase in DBP in the DT rats is not associated with cardiac alteration. Also these results strongly suggest that the increased BPV rather than increased DBP drove the PWV increase in DT compared with CT rats.

To document arterial stiffness modifications in response to long-term BPV, we chose to use young rats; an age during which the rise of BP and remodeling of the arterial wall is predominant. Indeed, it is well-established that the rise in BP and remodeling of the arterial wall of conduit arteries occur mostly between the 4th and 20th weeks in SHR. Therefore, the period of the present study (8th to 16th weeks of age) was suitable to document modifications in the structural and mechanical characteristics of the aorta in response to discontinuous antihypertensive treatment. There is a general consensus that PP provides indirect measure of arterial stiffness in isolated systolic hypertension (Benetos et al., 1997; Franklin et al., 1997). Indeed, PP is a strong predictor of arterial stiffness in hypertensive patients and in the general population, especially in the elderly. In our study, discontinuous administration of valsartan caused a significant decrease in telemetered PP despite the PWV increase compared with rats treated continuously with valsartan. We have already reported that in another experimental model of aortic stiffness, SHR treated with L-Name, PP is obviously not coupled with PWV and BP (Isabelle et al., 2015). In this model, the absence of a relation between PWV and PP was related to heart failure as reported in humans (Regnault et al., 2014). In our present day-to-day variability model, cardiac parameters were preserved (data not shown). Also, our model further demonstrates that a change in SBP, but not DBP, is the major determinant of PP increase in different aortic stiffening models (Guo et al., 2014).

After discontinuous treatment with valsartan, we found an increase in PWV associated with an enhancement of the medial cross-sectional area of the aorta and accumulation of fibronectin and its α_v_-integrins compared with CT rats. Whilst discontinuous treatment produced the same reduction in very short term BPV assessed by spectral analysis compared to continuous treatment, when short term variability was evaluated by ARV, DT was increased compared to continuous treatment. This augmentation of ARV occurred on the third day post treatment and not during the second day, when the blood pressure was the lowest. Thus, this increase in ARV seems closely related to the rise of blood pressure. Nevertheless, it may participate in the vascular alterations as already reported (Miao et al., 2003; Xie et al., 2003; Bouissou et al., 2014).

Thus, in the absence of a significant difference in SBP, day-to-day variability appears of the main mechanism to increase PWV after discontinuous treatment with valsartan. In addition, the specific accumulation of fibronectin found after discontinuous treatment compared with placebo treated rats, despite a significantly lower SBP also support the greater impact of day-to-day variability as opposed to BP in our model.

Apart from BP, the vascular remodeling process, especially the extracellular matrix composition, is considered as one of the major contributors to arterial stiffness. In the present study, collagen-to-elastin ratio, an index directly associated with the stiffness of the vascular wall, was unchanged within the different groups. Therefore, as in other experimental models of increased stiffness, accumulation of aortic fibronectin and its α_v_-integrin receptor appears to be a contributor to PWV in DT rats compared to CT ones (Bézie et al., 1998; Kakou et al., 2009; Bouissou et al., 2014). The absence of modification in both CD146 and the vWF, two biomarkers of endothelial dysfunction (Lagrange et al., 2014) indicates that the accumulation of the fibronectin observed in the present experiments results from its *in situ* synthesis by the extracellular matrix rather than an accumulation of the circulating form. *In vivo*, fibronectin matrix polymerization is a continuous process, with as much as 50% of the fibronectin matrix undergoing turnover every 24 h. By contrast, elastin and collagen are characterized by a very slow turnover (from weeks to years), fibronectin appears as the main protein playing a key role in the structural adaptation of the arterial wall in response to chronic hemodynamic stresses like BP or shear stress (Sehgel et al., 2015). Indeed, fibronectin is enhanced in a few animal models of increased arterial stiffness, whether BP level is increased or not (Lacolley et al., 2002; Bouissou et al., 2014). Nevertheless, the mechanism for PWV increase in the PT rats seems quite different, as the accumulation of fibronectin and its α_v_-integrin wasn't present in this group.

Several limitations of our study deserve consideration. First, hemodynamic parameters and PWV and arterial structure were investigated in two different sets of animals, excluding therefore the possibility to correlate day-to-day variability and aortic damage. Nevertheless, as animals received treatments in the same conditions, one could speculate that arterial structure and stiffness was comparable between the two sets of animals. Second, the groups of rats were relatively small. However, within the scope of chronic telemetery studies, these numbers were standard and our results were statistically significant and sufficient to highlight the role of BP variability on aortic stiffness.

## Conclusion

Discontinuous treatment by valsartan is able to induce chronic day-to-day BPV increase in SHR. In this model, we demonstrate that aortic stiffness, related to fibronectin accumulation, is jointly associated with BPV. Our results support the notion that BPV is a precursor to vascular stiffening. Also, this experimental model should pave the way for future experimental studies aimed at assessing how long-term variability might lead to an increase in aortic stiffness. Transposed to humans, the present results might have important clinical implications in the follow-up of hypertensive patients.

## Author contributions

CB: contribution to the design of the work, acquisition, analysis of data, and drafting the work. GL and SR: Interpretation of data, drafting the work. MS: Conception of the work and revising the work critically for important intellectual content. VR: contribution to the design of the work, acquisition, and analysis of biological data, interpretation of data and revising the work. YB: conception and design of the work, interpretation of data, drafting and revising the work. HD: Conception of the work, acquisition, and interpretation of hemodynamic data. Revising for important intellectual content. VM: Acquisition and analysis of immunohistological data, interpretation of data for the work, revising the work. All the authors have approved this version submitted for publication and agree to be accountable for all aspects of the work in ensuring that questions related to the accuracy or integrity of any part of the work are appropriately investigated and resolved.

### Conflict of interest statement

The authors declare that the research was conducted in the absence of any commercial or financial relationships that could be construed as a potential conflict of interest.

## Abbreviations

BP: blood pressure
BPV: blood pressure variability
CV: coefficient of variation
DBP: diastolic blood pressure
HR: heart rate
MCSA: media cross-sectional area
PWV: pulse wave velocity
SD: standard deviation
SBP: systolic blood pressure
SHR: spontaneously hypertensive rat
vWF: von Willebrand factor.
